# Supplementary material for: Stacking transfer of wafer-scale graphene-based van der Waals superlattices
Source: Nat Commun. 2023 Sep 6;14:5457. doi: 10.1038/s41467-023-41296-5 (PMC10482836; doi:10.1038/s41467-023-41296-5)
Supplement: Supplementary file 1 — Supplementary Information [file 41467_2023_41296_MOESM1_ESM.pdf]

Supplementary information for

Stacking transfer of wafer-scale graphene-based van der Waals superlattices

Guowen Yuan[1]†, Weilin Liu[1]†, Xianlei Huang[1], Zihao Wan[1], Chao Wang[1], Bing Yao[1], Wenjie Sun[2], Hang Zheng[1], Kehan Yang[1], Zhenjia Zhou[1], Yuefeng Nie[2], Jie Xu[1], and Libo Gao[1]*

[1] National Laboratory of Solid State Microstructures, Jiangsu Key Laboratory for Nanotechnology, School of Physics, Collaborative Innovation Center of Advanced Microstructures, Nanjing University, Nanjing, China.

[2] National Laboratory of Solid State Microstructures, Jiangsu Key Laboratory of Artificial Functional Materials, College of Engineering and Applied Sciences and Collaborative Innovation Center of Advanced Microstructures, Nanjing University, Nanjing, China.

† These authors contributed equally: Guowen Yuan; Weilin Liu

* Corresponding author e-mail: lbgao@nju.edu.cn

The supplementary information includes:

supplementary Fig. 1 to Fig. 14

Inventory of Supplementary Information:

Supplementary Fig. 1 | Characterization of the aqueous solution layer.

Supplementary Fig. 2 | Non-destructive transfer of monolayer graphene film.

Supplementary Fig. 3 | Comparison of the transferred graphene after different treatments.

Supplementary Fig. 4 | Raman spectra of the transferred monolayer graphene.

Supplementary Fig. 5 | Transport properties of the transferred monolayer graphene.

Supplementary Fig. 6 | Tuning the wetting angles of aqueous solution on graphene.

Supplementary Fig. 7 | PAT for cleaning the interface of double-layer graphene.

Supplementary Fig. 8 | Additional Raman spectra of transferred double-layer and triple-layer graphene.

Supplementary Fig. 9 | Single crystalline graphene film and the random stacking angles.

Supplementary Fig. 10 | Controlling the macroscopic stacking angle.

Supplementary Fig. 11 | Controlling the macroscopic stacking angle at 0°.

Supplementary Fig. 12 | Comparison of the macroscopical stacking angle and the twist angle.

Supplementary Fig. 13 | Additional transport properties of the stacking transferred double-layer and triple-layer graphene.

Supplementary Fig. 14 | Stacking transfer of graphene-based vdWS with other 2D materials.

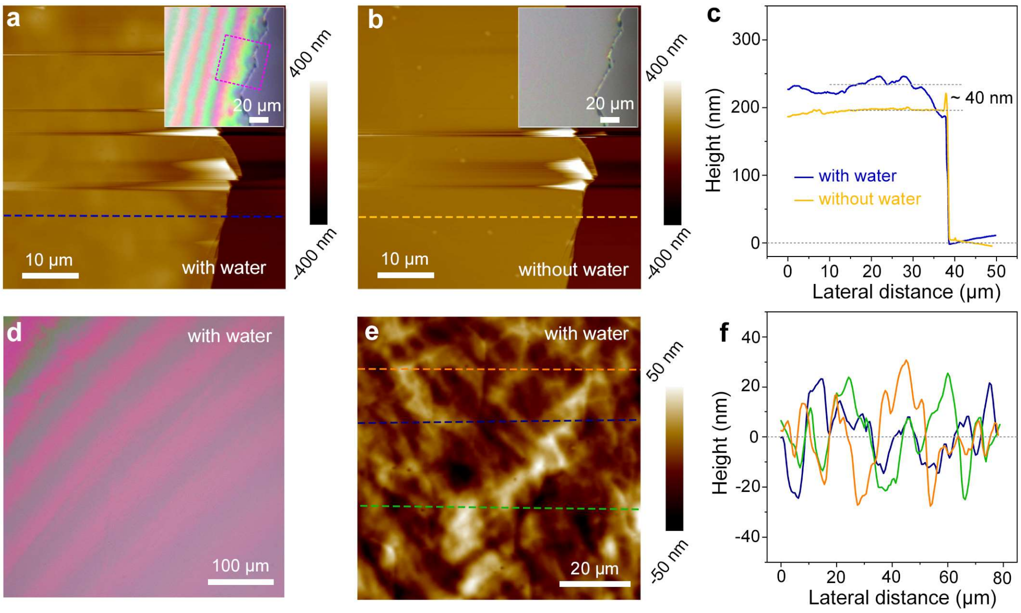

Supplementary Fig. 1 | Characterization of the aqueous solution layer. a, AFM height image of PMMA/aqueous solution/graphene on SiO_2/Si after slow spin process, inset is the corresponding optical image. **b**, *ex situ* AFM height image of the PMMA/graphene after fast spinning and baking processes, the aqueous solution is eliminated. **c**, Corresponding height profiles of the same position before and after removing aqueous solution, the height of the aqueous solution is about 40 nm. **d**, Typical large-sized optical image of PMMA/aqueous solution/graphene on SiO_2/Si. **e**, Typical large-sized AFM height image of PMMA/aqueous solution/graphene. **f**, Corresponding height profiles of the labels in **e**, the roughness fluctuation is ±25 nm.

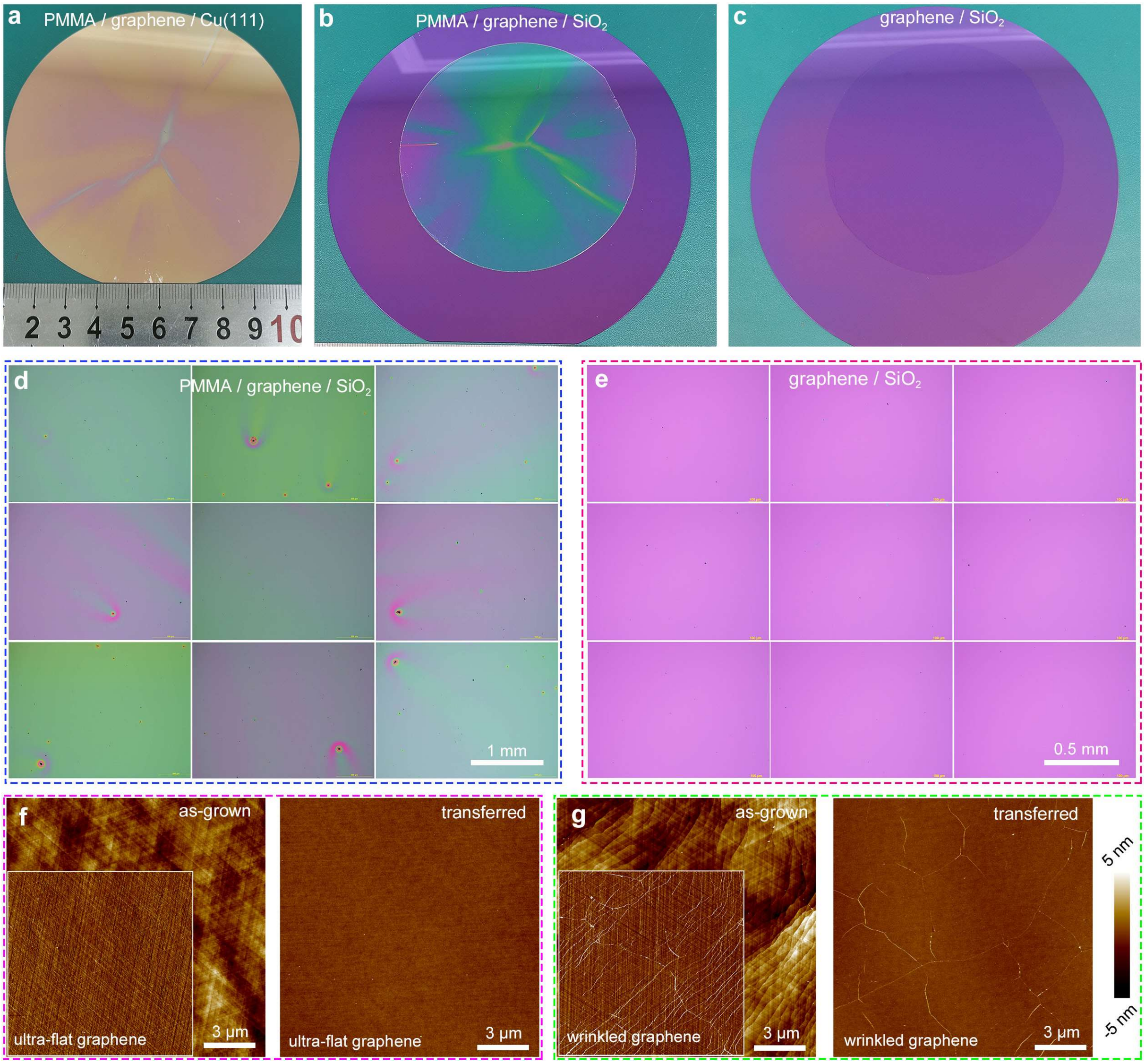

Supplementary Fig. 2 | Non-destructive transfer of monolayer graphene film. **a**, Photo of a 4-inch graphene film grown on Cu(111) covered by PMMA. **b**, Photo of the graphene film in **a** transferred to a 6-inch SiO_2/Si wafer. **c**, Photograph the transferred 4-inch graphene film in **b** after removing PMMA. **d**, Typical optical image captured from **b**, there are no folds formed after the spin process. **e**, Typical optical image captured from **c**, there are no folds and cracks after removing PMMA. **f**, *ex situ* AFM images of the as-grown ultra-flat graphene film on Cu(111) and as-transferred films on SiO_2/Si. **g**, *ex situ* AFM images of the as-grown wrinkled graphene on Cu(111) and as-transferred films on SiO_2/Si. Inset in **f** and **g** is the corresponding phase image. Both **f** and **g** show that the original morphology of graphene films can be well preserved without any damage.

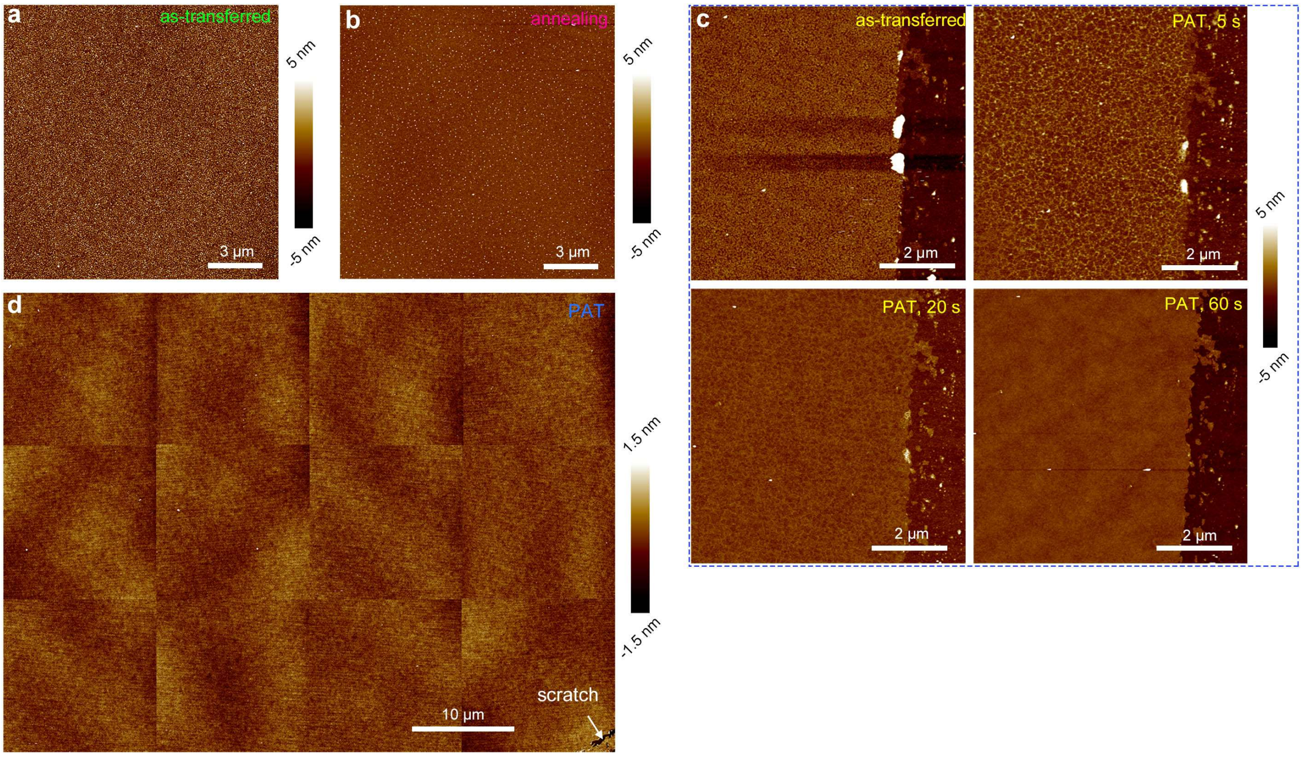

Supplementary Fig. 3 | Comparison of the transferred graphene after different treatments. a, Typical AFM height images of as-transferred graphene films, there are many residual NPs on the surface. **b**, Typical AFM height images of graphene films after vacuum annealing, there are still some residual NPs on the surface. **c**, *ex situ* AFM images of the graphene film after different PAT process: as-transferred; after PAT process of 15 W, 5 s; after PAT process of 15 W, 20 s; after PAT process of 15 W, 60 s. **d**, Stitched large-sized AFM height images of graphene films after PAT process, the surface is clean and there are no NPs, wrinkles and folds.

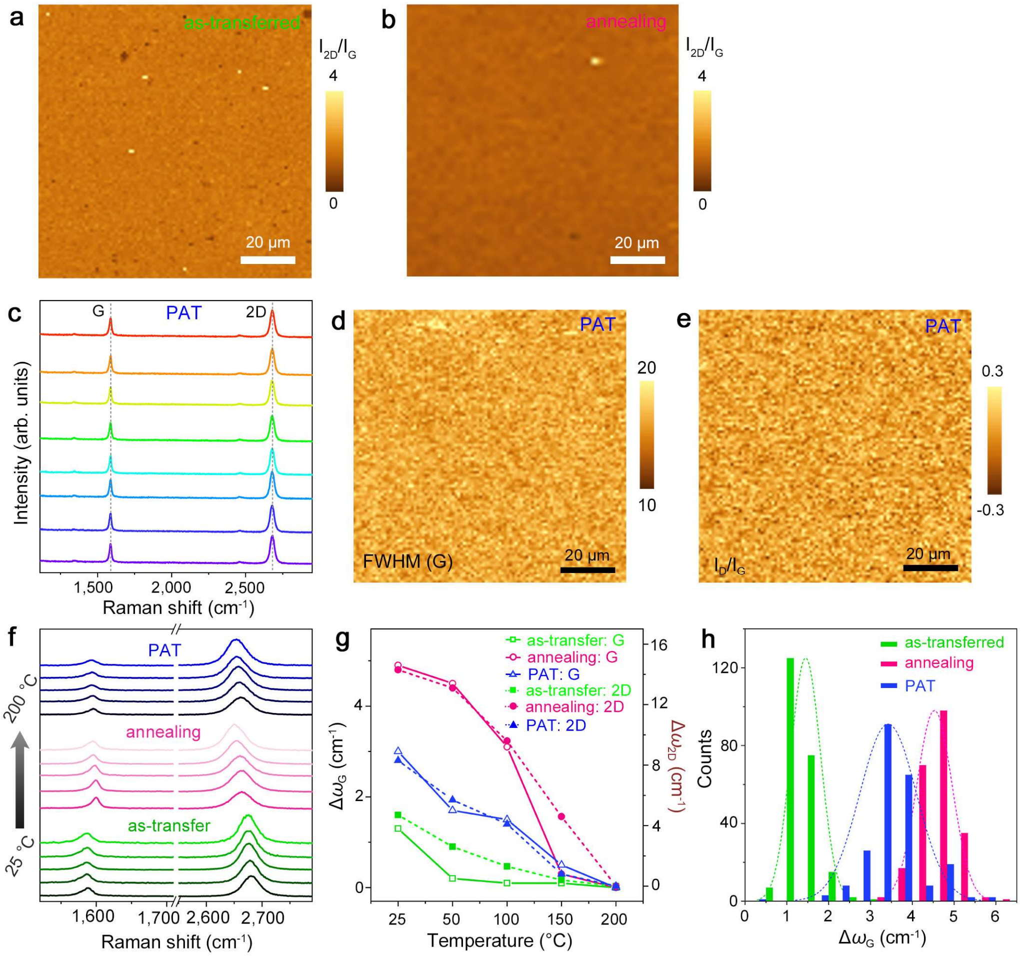

Supplementary Fig. 4 | Raman spectra of the transferred monolayer graphene. **a**, Raman mapping of I_{2D}/I_G of the as-transferred graphene in the region of 100 × 100 μm^2. **b**, Raman mapping of I_{2D}/I_G of the vacuum annealed graphene in the region of 100 × 100 μm^2. **c**, Typical Raman spectra of the transferred monolayer graphene after PAT process, measured on the random position. **d**, Raman mapping of FWHM of G band in the region of 100 × 100 μm^2, showing the homogeneity after PAT process. **e**, Raman mapping of I_D/I_G in the region of 100 × 100 μm^2, showing the homogeneity and high quality after PAT process. **f**, *in situ* variable temperature Raman spectra for the as-transferred graphene, vacuum annealed graphene and PAT graphene films on SiO_2/Si wafers, the measurement temperature is from 25 °C to 200 °C. **g**, Extracted $\Delta\omega_G$ and $\Delta\omega_{2D}$ at different temperature, the as-transferred graphene is the most weakly coupled with the substrate, and the annealed graphene is the most strongly coupled with the substrate. **h**, Statistic plots of $\Delta\omega_G$ of different graphene films from 25 °C to 200 °C, indicating PAT process can effectively reduce the coupling between graphene and substrate.

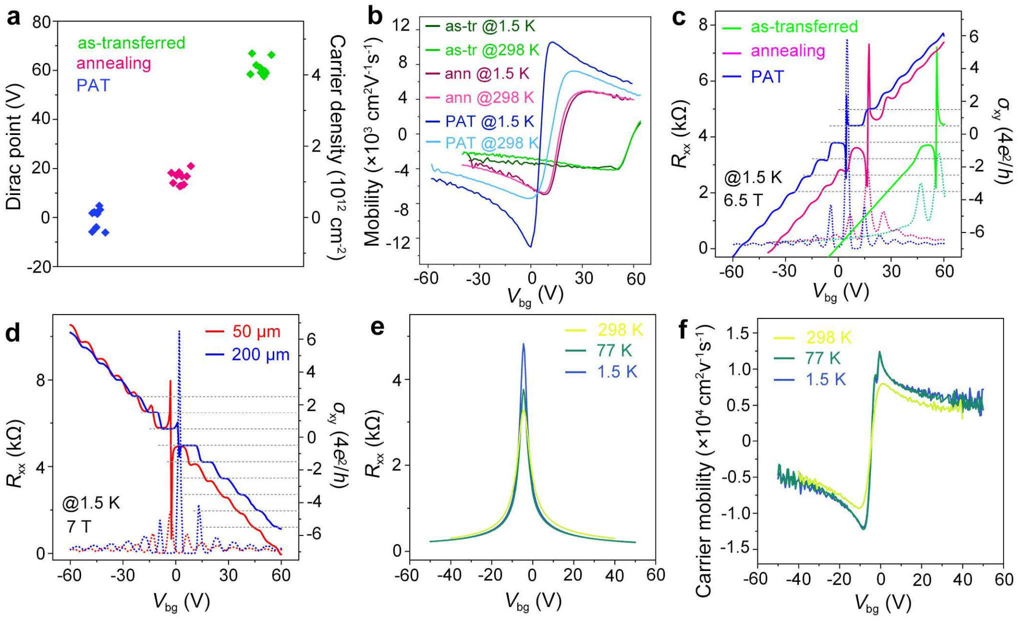

Supplementary Fig. 5 | Transport properties of the transferred monolayer graphene. **a**, Dirac point and the corresponding carrier density of thirty FET devices fabricated by the as-transferred, annealing and PAT graphene films, respectively. The PAT graphene shows the lowest doping concentration. **b**, Extracted FET carrier mobility of the graphene in main Figure 2g at 1.5 K and RT (298 K). The PAT decoupling graphene shows the highest mobility. **c**, R_{xx} and σ_{xy} of the as-transferred, annealing and PAT graphene films at temperature of 1.5 K and under B⊥ of 6.5 T. **d**, R_{xx} and σ_{xy} of PAT graphene with the linewidths of 50 μm and 200 μm at temperature of 1.5 K and under B⊥ of 7 T, they show no obvious difference. **e**, Transport behaviours of FET devices fabricated by the PAT graphene with 1 mm linewidth at RT, 77 K and 1.5 K, respectively. **f**, Extracted FET carrier mobility from the data of **e,** the hole mobilities are ~12,000 $cm^2V^{-1}s^{-1}$, ~12,100 $cm^2V^{-1}s^{-1}$ and ~9,300 $cm^2V^{-1}s^{-1}$ for 1.5 K, 77 K and RT, and the electron mobilities are 12,400 $cm^2V^{-1}s^{-1}$, 12,300 $cm^2V^{-1}s^{-1}$ and 8,000 $cm^2V^{-1}s^{-1}$ for 1.5 K, 77 K and RT, respectively.

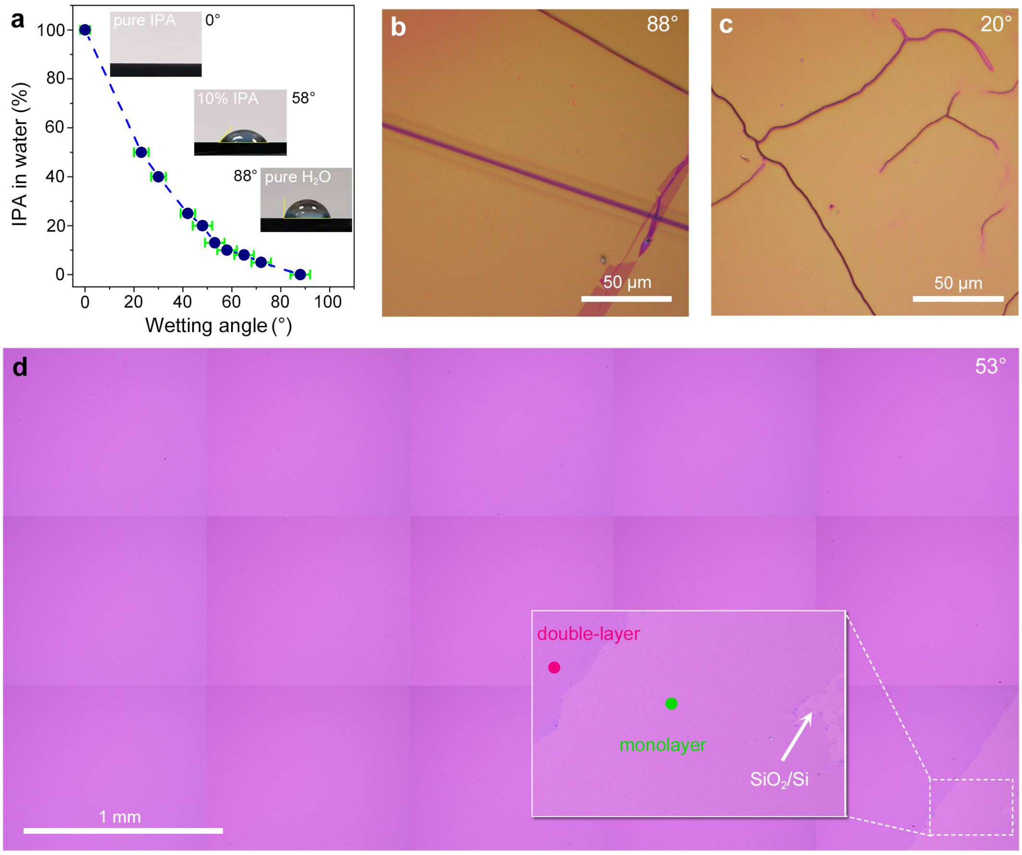

Supplementary Fig. 6 | Tuning the wetting angles of aqueous solution on graphene. a, Wetting angles of DI water on graphene by adding different proportions of IPA inside. Insets are the captured photos of wetting angles on graphene. **b**, Typical optical image of the transferred PMMA/ graphene/ graphene films obtained with the wetting angle of 88°, there are significant folds formed. **c**, Typical optical image of the transferred PMMA/graphene/graphene films obtained with the wetting angle of 20°, the relatively short but abundant folds are still formed. **d**, Optical image of the transferred double-layer graphene films with the wetting angle of 53°, it is homogenous on large scale and only the scratches show the existence of graphene.

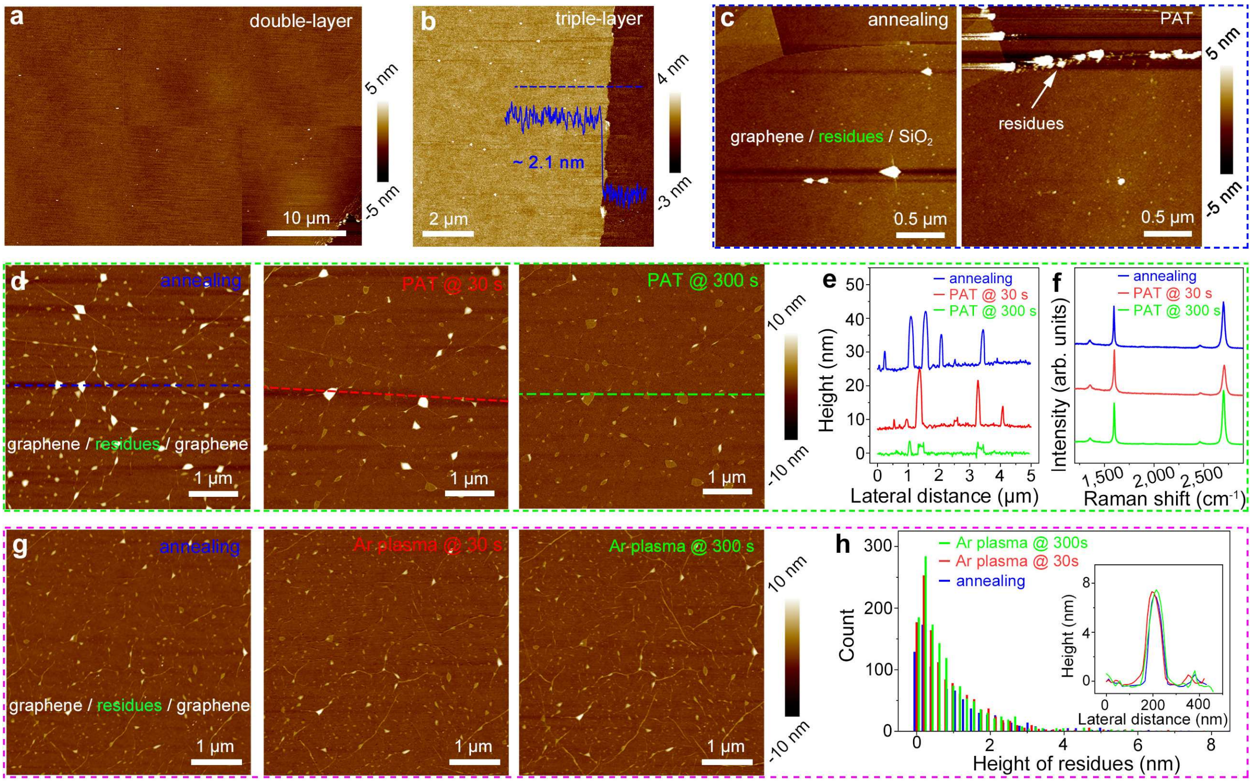

Supplementary Fig. 7 | PAT for cleaning the interface of double-layer graphene. a, Large-sized AFM image of the transferred double-layer graphene, there are almost no folds and no cracks formed. **b**, Large-sized AFM image of the transferred triple-layer graphene, there are almost no folds formed and the height of the triple-layer films is ~2.1 nm. **c**, *ex situ* AFM images of the transferred monolayer graphene with residues trapped in the interlayer between graphene and SiO_2/Si, indicating that the trapped residues decrease after PAT and will be excluded from the graphene edges. **d**, *ex situ* AFM images of the transferred double-layer graphene with residues trapped in the interlayers after the continuous treatments: vacuum annealing, PAT for 30 s and PAT for 300 s. **e**, Height profiles of the same residues taken from the corresponding lines in **d**, indicating PAT realizes cleaning the interlayer and flattening the surface. **f**, Typical Raman spectra of double-layer graphene after the treatment in **d**, showing that the double-layer graphene is non-destructive during the transfer and PAT process. **g**, *ex situ* AFM image of the transferred double-layer with residues trapped in the interlayers after continuous treatments: vacuum annealing, Ar plasma for 30 s and Ar plasma for 300 s. **h**, Statistic plot of residue trapped in the interlayers from the data of **g**, inset is the height profile of the same residues in **g** and shows that Ar plasma is not helpful for cleaning the interlayer of double-layer graphene films.

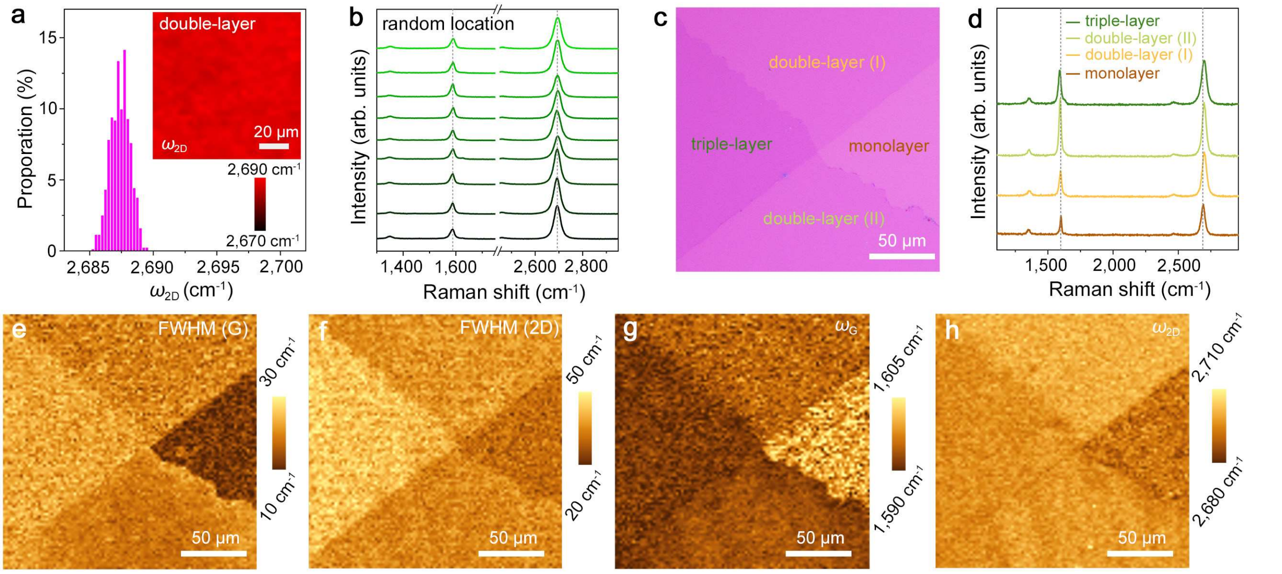

Supplementary Fig. 8 | Additional Raman spectra of transferred double-layer and triple-layer graphene. a, Statistical distribution of ω_{2D} in the region of 100 × 100 μm^2, inset is the corresponding Raman mapping and shows the homogenous distribution of peak positions. **b**, Raman spectra collected randomly from a transferred wafer-scale double-layer graphene, indicating the homogeneity of the film. **c**, Optical image of the transition region containing monolayer, double-layer and triple-layer graphene. **d**. Typical Raman spectra measured from the regions of different layer number in **c**. **e**-**h**, Raman mapping of the same regions in **c**, the images from left to right are the FWHM of G band, FWHM of 2D band, ω_G and ω_{2D}, respectively. They show that the graphene films with different layer number are all homogenous, and the difference between the two double-layer regions is caused by the different twist angles during the stacking transfer process.

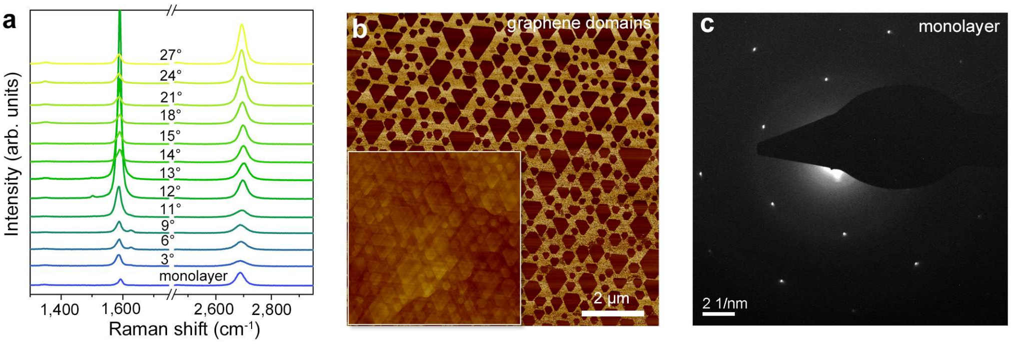

Supplementary Fig. 9 | Single crystalline graphene film and the random stacking angles. a, Typical Raman spectra of the transferred double-layer graphene with different twist angles if without specific manipulation. **b**, AFM phase image of graphene grains grown on Cu(111) substrate, inset is the corresponding AFM height image. The orientations of individual grains before joining into the film are uniformly distributed. **c**, Typical SAED pattern of monolayer graphene film.

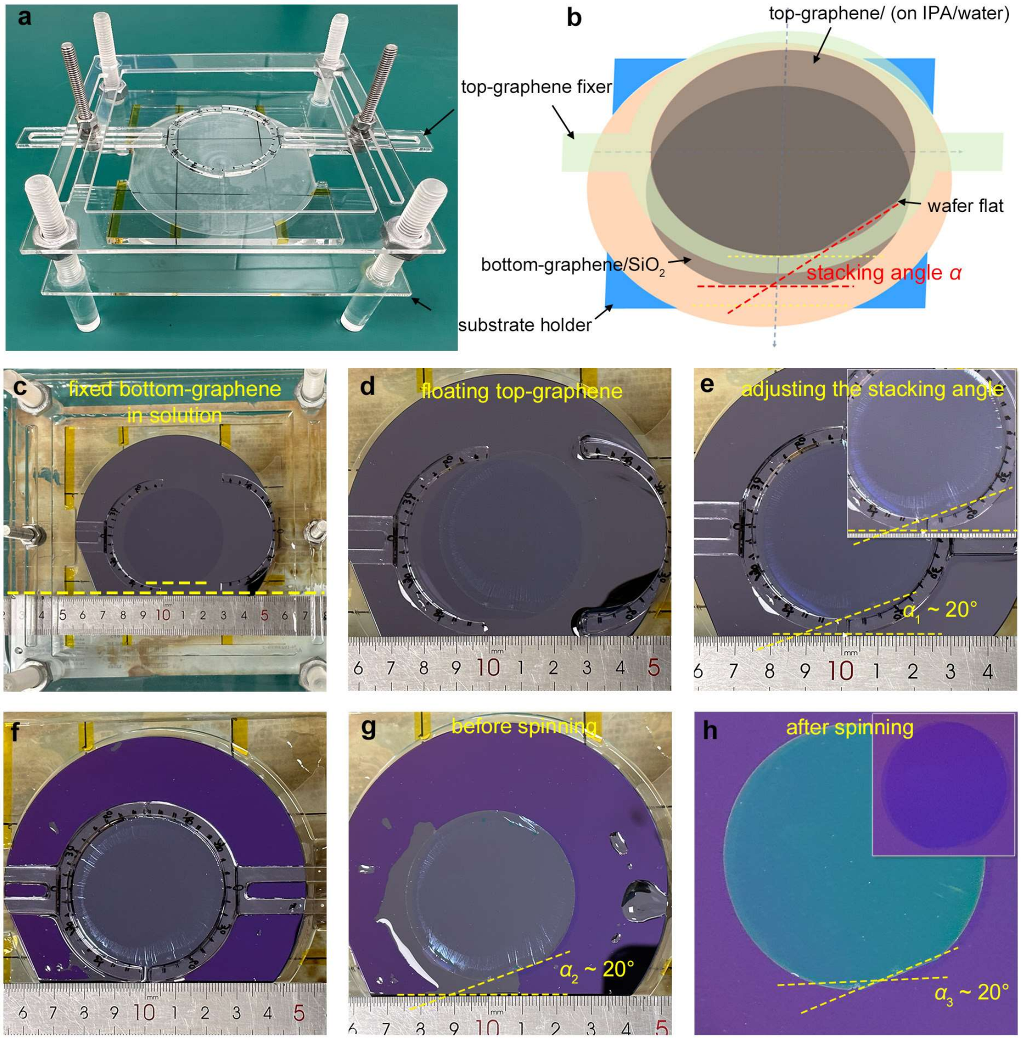

Supplementary Fig. 10 | Controlling the macroscopic stacking angle. a, Photo of the customized transfer operation system, consisting of a water basin and a rotation controller. The as-transferred graphene (bottom graphene) is placed on the substrate holder, the rotation controller is used to fix the top graphene, which is floating on the aqueous solution. **b**, Schematic of the angle controlling principle, the macroscopic stacking angle is regulated by the wafer flats of graphene. **c**, Photo of the bottom graphene immersed in the solution with the adjusted wafer flat parallel to the ruler. **d**, Top graphene floating on solution without any control. **e**, Fixed top graphene and adjusting their twist angle α_1, here α_1 is set at ~20°. **f**, Exhausting the solution and descend the top-graphene, then finishing an initial alignment between the two graphene films. **g**, Double-layer graphene films after removing the fixer and before spinning, the macroscopic twist angle remains unchanged, $\alpha_2 = \alpha_1 = $ ~20°. **h**, Double-layer graphene films after spinning-assisted transfer process, the top graphene is nearly not rotated compared to the bottom layer during the spinning process and the final macroscopic twist angle α_3 still remain unchanged. Inset is the double-layer graphene films after removing PMMA.

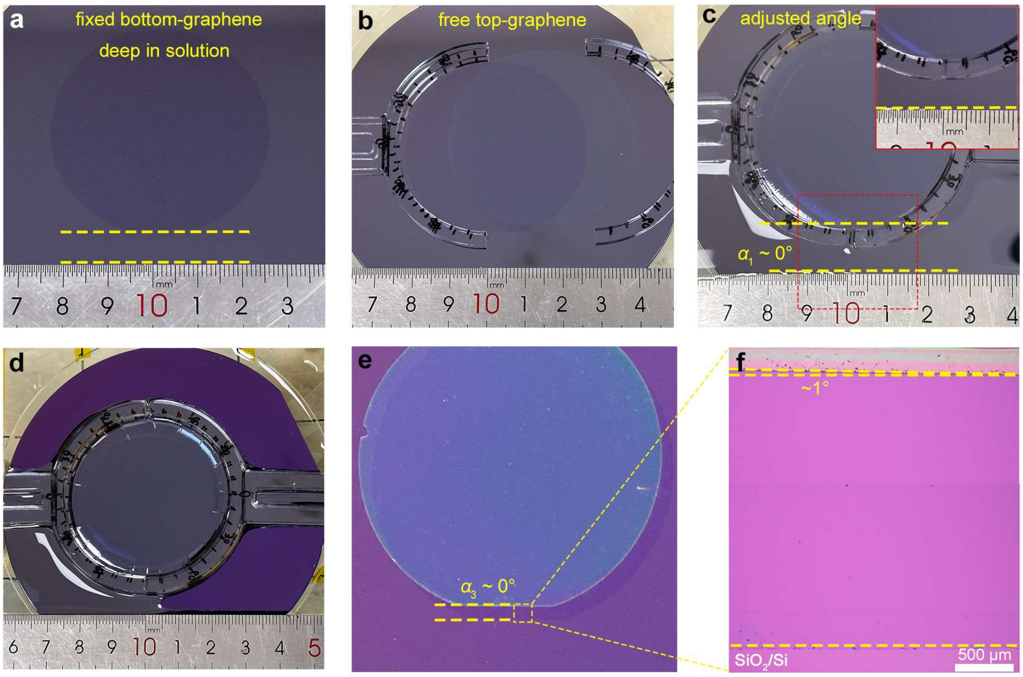

Supplementary Fig. 11 | Controlling the macroscopic stacking angle at 0°. a, Photo of the bottom graphene immersed in aqueous solution and the wafer-flat of graphene adjusted parallel to the ruler. **b**, Top PMMA/graphene floating on aqueous solution without any control. **c**, Adjusting the macroscopic twist angle at approximately 0°. **d**, Exhausting the solution to descend the top-graphene and finally achieve an initial alignment between the two graphene films. **e**, Graphene films after spinning-assisted transfer process, photo is captured by camera showing that the as-obtained twist angle still remains at ~0°. **f**, High resolution optical image shows that the twist angle is about 1°.

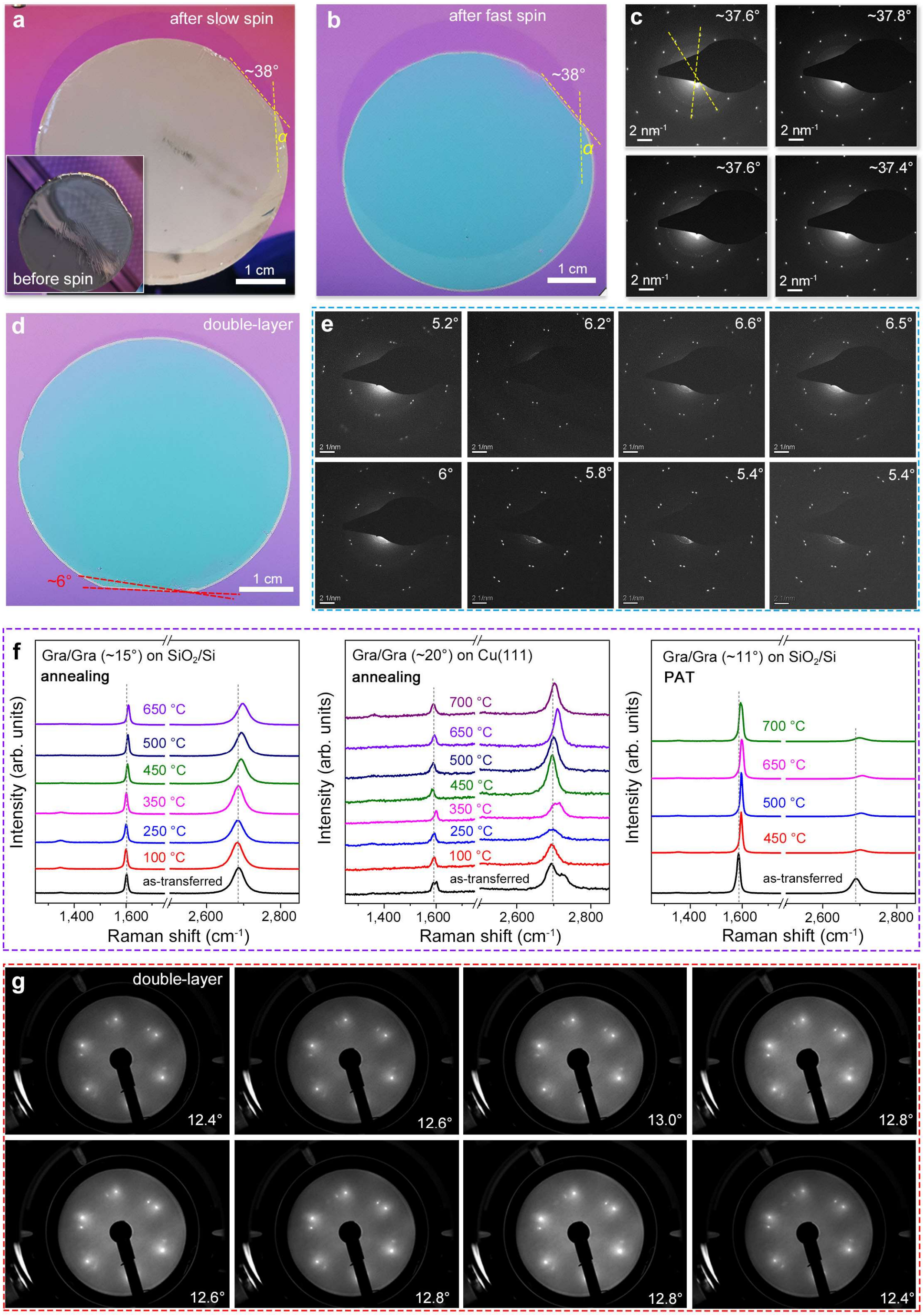

Supplementary Fig. 12 | Comparison of the macroscopical stacking angle and the twist angle. a, Photo of stacking double-layer graphene films after the slow spinning process, the macroscopic stacking angle between the double-layer films is controlled at ~38° according to the graphene wafer flats. Inset is the corresponding photo before the slow spinning process and there is a thick solution layer between the two films. **b**, Photo of stacked double-layer graphene after the fast spinning process, the macroscopic stacking angles remain unchanged at ~38°. **c**, Corresponding SAED patterns, the twist angles measured by SAED are basically consistent with the macroscopic stacking angle from graphene wafer flats. **d**, Photo

of a 2-inch double-layer graphene film with the macroscopic stacking angle of ~6° measured from graphene wafer flats. **e**, Representative SAED patterns of the double-layer graphene film in **d**, the twist angles of double-layer graphene coincide with the macroscopic stacking angle. **f**, *ex situ* Raman spectra of double-layer graphene after annealing at different temperature, left is the as-transferred double-layer graphene on SiO_2/Si, middle is the as-transferred double-layer graphene on Cu(111) and right is the PAT double-layer graphene on SiO_2/Si. **g**, LEED patterns of a transferred double-layer graphene films on Cu(111) obtained from different positions, the electron beam energy is 170 eV and the spot size is about 1 mm, indicating the twist angles are homogenous in large scale.

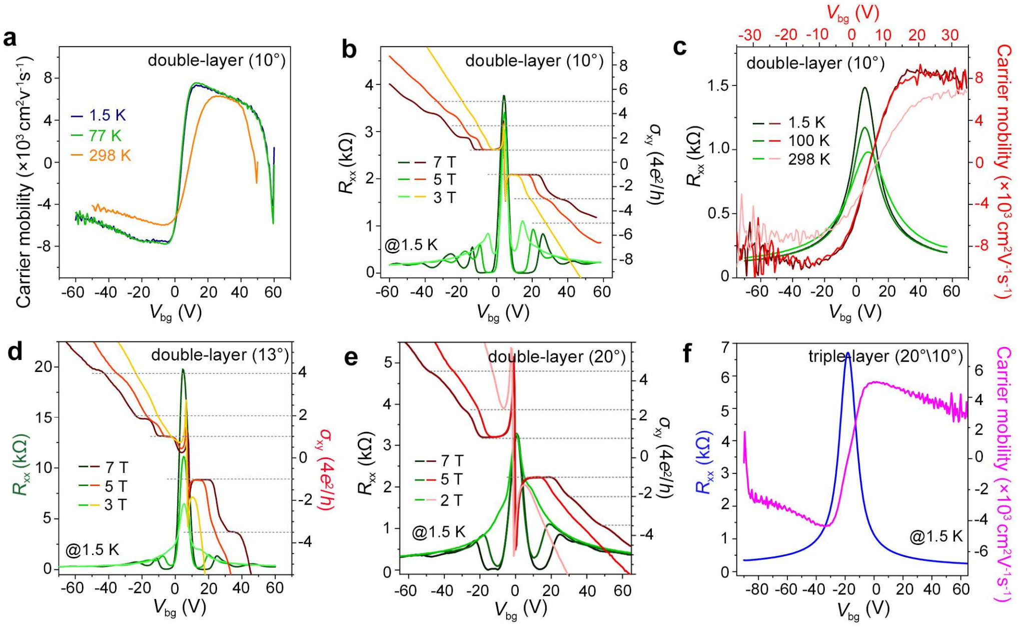

Supplementary Fig. 13 | Additional transport properties of the stacking transferred double-layer and triple-layer graphene. a, Extracted hole mobility of the FET devices fabricated by double-layer graphene films with twist angle of ~10° at 1.5 K, 77 K and RT, the hole mobilities are 7,500 $cm^2V^{-1}s^{-1}$ and 5,950 $cm^2V^{-1}s^{-1}$ at 1.5 K and RT, and the electron mobilities are 7,300 $cm^2V^{-1}s^{-1}$ and 6,250 $cm^2V^{-1}s^{-1}$ at 1.5 K and RT, respectively. **b**, R_{xx} and σ_{xy} of the same double-layer graphene in **a**, measured at the temperature of 1.5 K and under B⊥ of 3 T, 5 T and 7 T, respectively. **c**, Typical transport curves and the extracted carrier mobility of the double-layer graphene with twist angle of ~13°, the hole mobilities are ~10,000 $cm^2V^{-1}s^{-1}$ and ~7,100 $cm^2V^{-1}s^{-1}$ at 1.5 K and RT, and the electron mobilities are ~8,700 $cm^2V^{-1}s^{-1}$ and ~6,200 $cm^2V^{-1}s^{-1}$ at 1.5 K and RT, respectively. **d**, R_{xx} and σ_{xy} of double-layer graphene with twist angle of ~13°, measured at the temperature of 1.5 K and under B⊥ of 3 T, 5 T and 7 T, respectively. **e**, R_{xx} and σ_{xy} of double-layer graphene with twist angle of ~20°, measured at the temperature of 1.5 K under B⊥ of 2 T, 5 T and 7 T, respectively. **f**, R_{xx} of triple-layer graphene with the twist angles of 20° (1st and 2nd) and 10° (2nd and 3rd) as a function of V_{bg} at 1.5 K and extracted mobility, the electron mobility reaches ~4,950 $cm^2V^{-1}s^{-1}$ at 1.5 K.

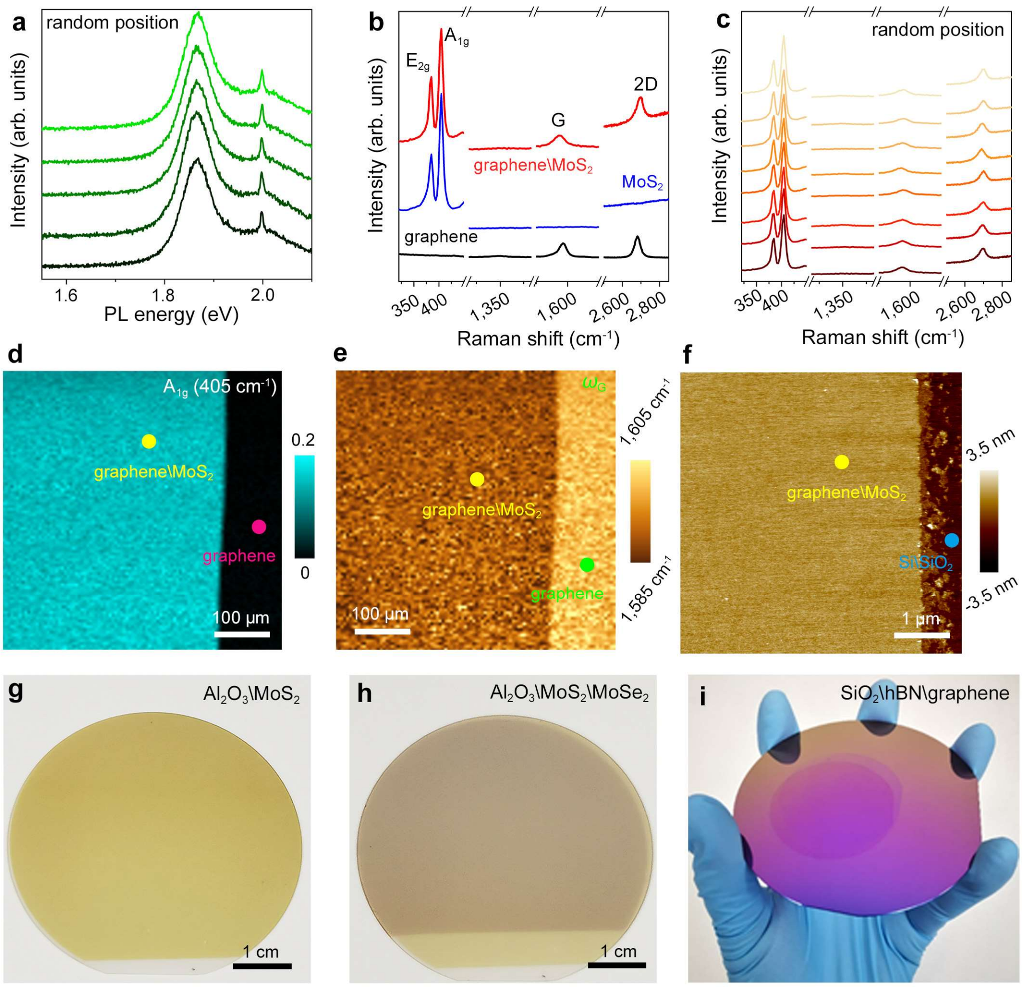

Supplementary Fig. 14 | Stacking transfer of graphene-based vdWS with other 2D materials. a, Typical PL spectra of graphene\$MoS_2$ on Si\$SiO_2$. **b**, Typical Raman spectra of individual MoS_2, individual graphene and transferred graphene\$MoS_2$ heterostructure. **c**, Raman spectra collected at random positions of the same graphene\$MoS_2$. **d**, Raman intensity mapping of MoS_2 A_{1g} band (405 cm^{-1}) in graphene\$MoS_2$ heterostructure across the region of 500 × 500 μm^2. **e**, Raman mapping of graphene ω_G in the same graphene\$MoS_2$ across the region of 500 × 500 μm^2. **f**, Typical AFM image of the graphene\$MoS_2$ heterostructure. **g**, Photo of the transferred 2-inch MoS_2 film on Al_2O_3 substrate. **h**, Photo of the transferred 2-inch MoS_2\$MoSe_2$ on Al_2O_3 substrate. **i**, Photo of the transferred 2-inch hBN\graphene on 4-inch Si\$SiO_2$ wafer.
